# Cross-Model Uncertainty-Aware Minimal Editing of Cis-Regulatory Elements for Cell-Type-Selective Design

**DOI:** 10.3390/genes17091062

**Published:** 2026-09-01

**Authors:** Angran Xia, Changwei Wang, Yemao Xia

**Affiliations:** 1 School of Chemistry and Chemical Engineering, Shaanxi Normal University, 620 West Chang’an Avenue, Chang’an District, Xi’an 710119, China; 2 College of Science, Nanjing Forestry University, 159 Longpan Road, Xuanwu District, Nanjing 210037, China

**Keywords:** cis-regulatory elements, cell-type specificity, regulatory-sequence design, functional genomics, machine learning, reporter screening

## Abstract

**Background/Objectives:** Cis-regulatory elements (CREs) help mediate cell-type-selective gene expression, yet sequence-to-activity models are rarely converted into compact, experimentally tractable variant panels that retain natural regulatory scaffolds. We developed SafeEdit-CRE to select minimal edits that preserve cross-model specificity while reducing predictive uncertainty, yielding an auditable CRE variant library for reporter-screening prioritization. **Methods:** SafeEdit-CRE combines the published Malinois sequence–activity predictor with an architecture-distinct reviewer ensemble, validation-calibrated uncertainty, sequence-domain controls, and constrained beam search. Natural 200 nt CREs were edited under fixed substitution budgets of 1, 5, 10, or 20 nucleotides and evaluated by a separately trained multi-kernel ensemble kept entirely separate from candidate generation. **Results:** Across 600 held-out CRE parents in K562, HepG2, and SK-N-SH cells, SafeEdit-CRE achieved a mean cross-model specificity-margin gain of 0.877 versus 0.822 for greedy editing (paired difference 0.055; 95% CI 0.040–0.071). Relative to a compute-matched primary-model beam, SafeEdit-CRE yielded a similar cross-model specificity-margin gain (difference −0.004; 95% CI −0.011–0.004) with lower ensemble uncertainty (0.314 vs. 0.335). In a locked 90-parent benchmark, 60.6% of designs passed all nine prespecified sequence and model-agreement checks, compared with 38.8% for greedy and 13.1% for random substitution (paired difference 21.8 percentage points; 95% CI 18.3–25.3). **Conclusions:** SafeEdit-CRE reduced 3240 designs to 74 Tier A computational candidates prioritized for reporter screening across three cell types and four edit budgets. By separating search from cross-model audit and enforcing explicit edit budgets and sequence–domain safeguards, the framework provides a reproducible and auditable approach to computational CRE prioritization, regulatory-grammar studies, and synthetic regulatory-element design.

## 1. Introduction

Cis-regulatory elements (CREs) encode when and where genes are expressed through transcription-factor binding sites and their surrounding sequence context [1,2,3,4,5]. CREs are short enough to synthesize, and their activity can be measured directly. Massively parallel reporter assays (MPRAs), STARR-seq, and saturation-mutagenesis assays now quantify the effects of thousands to hundreds of thousands of regulatory sequences and variants in parallel [6,7,8,9,10,11]. These data have supported a progression from early convolutional and hybrid sequence models to long-range architectures and models that design synthetic enhancers [12,13,14,15,16,17,18,19,20,21].

Yet translating these predictive models into experimentally tractable CRE variant panels remains a practical bottleneck for regulatory genomics and its downstream applications. Most design strategies return either unconstrained high-scoring sequences or de novo enhancers, making it difficult to connect the model’s internal logic to the modular grammar of natural regulatory elements—and even harder to prioritize candidates that will validate in follow-up experiments. When the goal is to understand how local sequence changes alter cell-type-specific output or to engineer tissue-specific regulatory elements for gene therapy, synthetic biology, or functional interpretation of disease-associated noncoding variants, what is needed instead is a compact set of interpretable edits anchored to a natural CRE scaffold, with built-in safeguards that reduce the fraction of designs that fail experimental validation.

Most regulatory-design studies have emphasized de novo generation or direct score maximization [21,22,23]. A different problem arises when the starting point is a natural regulatory element and only a few substitutions are acceptable. We call this setting minimal editing: the aim is to increase cell-type selectivity while retaining nearly all of the parent CRE. The natural parent supplies the sequence context, and the edit budget fixes both design distance and synthesis burden. Each proposed variant can, therefore, be interpreted as a controlled perturbation of an existing regulatory scaffold.

A single-predictor search can assign its highest scores to sequences that exploit model-specific behavior, even when the predictor performs well on held-out natural sequences [24,25,26,27]. Greedy search is especially exposed because every substitution is retained on the basis of its immediate gain under one objective. A wider beam samples more edit paths but still ranks them with the same model. The unresolved problem is selection: identifying a small set of interpretable edits whose predicted effects persist across models. Separately trained review and calibrated uncertainty give direct criteria for making that selection.

**Figure 1:**
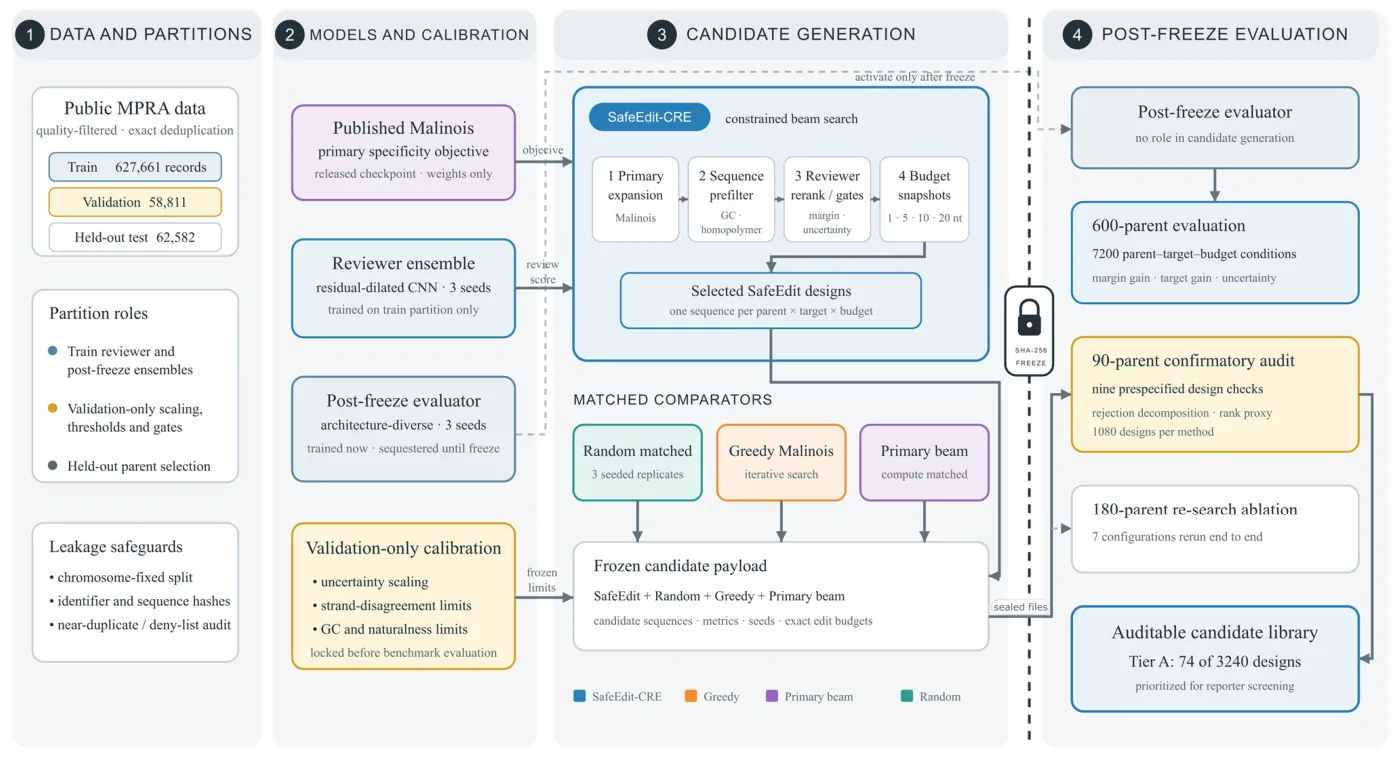
SafeEdit-CRE study design. A four-stage workflow: data and partitions (public MPRA, train/validation/held-out test, leakage safeguards), model development and calibration (published Malinois primary objective, reviewer ensemble, post-freeze evaluator, validation-only thresholds), candidate generation (SafeEdit-CRE constrained beam search with primary expansion, sequence prefilter, reviewer rerank/gates and budget snapshots alongside random matched, greedy Malinois and a compute-matched primary beam), and post-freeze evaluation (600-parent endpoints, 90-parent nine-check audit, 180-parent re-search ablation, Tier A library). Candidate files are sealed by a SHA-256 freeze boundary before post-freeze evaluation.

We developed SafeEdit-CRE to address this problem (Figure 1). The published Malinois predictor [21] provides the primary cell-type specificity objective. An architecture-diverse residual-dilated CNN ensemble serves as a reviewer during constrained beam search, while validation-calibrated uncertainty and sequence-domain controls (strand consistency, GC shift, homopolymers, 6-mer naturalness) limit sequence–domain deviations and flag potentially out-of-domain edits. Selected sequences are then assessed by a second, separately trained multi-seed ensemble that is held out from the search process, providing an additional layer of cross-model evaluation. Fixed edit budgets of 1, 5, 10, and 20 nucleotides allow the effect of design freedom to be quantified without conflating minimal editing with unconstrained sequence generation.

We benchmark SafeEdit-CRE against three comparators: random substitutions as a null baseline, iterative greedy optimization to test whether local score maximization is sufficient, and a compute-matched primary-model beam that uses the same beam width and expansion schedule as SafeEdit but only the primary predictor score. The compute-matched beam is critical because it isolates the benefit of cross-model selection from the benefit of a larger candidate pool. We evaluate four complementary endpoints: cross-model generalization on 600 held-out natural CRE parents across K562, HepG2, and SK-N-SH; a nine-criterion design-quality benchmark on 90 non-overlapping confirmatory parents; stratified analyses by cell type and edit budget; and complete re-search ablations that identify which components govern the trade-off between predicted selectivity and uncertainty.

SafeEdit-CRE increased cross-model specificity margin over greedy editing and matched a compute-matched beam on predicted specificity while selecting variants with lower uncertainty. In the locked design-quality benchmark, the pass rate increased from 38.8% to 60.6%. The final Tier A panel contains 74 sequences distributed across the three cell types and all four edit budgets, providing a focused library for reporter screening and subsequent analysis of cell-type-selective regulatory grammar.

## 2. Materials and Methods

### 2.1. MPRA Data and Fixed Chromosome Partitions

We used the public MPRA sequence-performance table from Gosai et al. [21], containing activity estimates and standard errors for K562, HepG2, and SK-N-SH. The processed activity data are provided in Supplementary Table S2 of that publication and archived at Zenodo record 10698014 (https://zenodo.org/records/10698014, accessed on 26 August 2026); the Malinois model checkpoint is released at https://github.com/harrisonliba/Malinois, accessed on 26 August 2026. The upstream chromosome partition was preserved: chromosomes 19, 21, and X were validation; chromosomes 7 and 13 were test; remaining eligible chromosomes were training. Records were required to contain only A/C/G/T, have length at most 200 nt, contain finite activity and standard-error values, and have maximum reported log-fold-change standard error below 1.0. Exact sequence duplicates were removed before full-data model training. Sequences shorter than 200 nt were centered in a 200-position tensor, and a fifth input channel marked valid positions.

### 2.2. Leakage and Parent-Overlap Auditing

We examined exact identity, reverse-complement identity, primary-variant group membership, and cross-split Hamming distance up to three among 200 nt constructs. The near-duplicate audit identified 27 cross-split pairs involving eight training-side variant groups. Parent deny lists included earlier development and confirmation parents, matching identifiers, sequence hashes, and audit-flagged conflicts. The 600-parent primary-evaluation table was created without activity labels by sorting eligible 200 nt test sequences by SHA-256 of ID + tab + sequence and selecting the first 600 after deny-list removal. The ablation study used a fixed 180-parent subset, and the nine-criterion benchmark used a fixed 90-parent confirmatory subset after six nominal parents were found to overlap with an earlier exploratory benchmark and were excluded by identifier without reference to outcomes. Parent overlap across phases was checked by identifier and sequence hash.

The full-data loader performed exact deduplication. The separate Hamming-distance manifest was applied to parent eligibility, ensuring that no flagged near-duplicate was admitted to the editing benchmarks.

### 2.3. Primary Predictor

Malinois BassetBranched, released with Gosai et al. [21], served as the primary predictor. The checkpoint was loaded in weights-only mode. Each sequence was evaluated in forward and reverse-complement orientations, and the activity prediction was averaged. For target cell t, the specificity margin was

$$M_{t}(x)=f_{t}(x)-\underset{j\neq t}{max}f_{j}(x),$$

where $f_{j}(x)$ is predicted activity in cell type j. Candidate margin gain was $\Delta M_{t}=M_{t}(x_{edit})-M_{t}(x_{parent})$.

### 2.4. Reviewer and Cross-Model Evaluation Ensembles

Two architecture-diverse heteroscedastic CNNs were trained on the quality-filtered training partition of the Gosai et al. MPRA table. After filtering, the dataset comprised 749,054 records (627,661 training, 58,811 validation, 62,582 test) under the upstream chromosome partition (chr 19/21/X validation; chr 7/13 test; remaining chromosomes training). Both ensembles were trained exclusively on the 627,661 training-split records; checkpoints were selected by validation-split mean Pearson correlation (early stopping, patience six), and the 62,582-record test split was never used to choose checkpoints, architectures, hyperparameters, or variance scales (Supplementary Table S11). The reviewer ensemble was a residual-dilated 1D CNN with an 11 nt stem, residual blocks at dilations 1, 2, 4, and 8, and shared mean and log-variance heads; it was available during candidate search for cross-model reranking. The cross-model evaluation ensemble (termed the post-freeze evaluator in the procedural protocol below) was a multi-kernel CNN with parallel 3, 7, 15, and 21 nt branches, pointwise fusion, depthwise convolution, and separate mean and variance heads [28,29]. The use of seeded ensembles and predictive variance follows established neural-network uncertainty strategies [29,30,31]. Each architecture used three fixed seeds: 20260721–20260723 for the reviewer and 20260731–20260733 for the cross-model evaluation ensemble.

Models were trained for, at most, 20 epochs with AdamW [32], cosine learning-rate decay, batch size 256, learning rate $10^{-3}$, weight decay $10^{-4}$, dropout 0.25 [33], and gradient clipping at 5.0. The loss combined heteroscedastic Gaussian negative log likelihood, reported measurement standard error, and a reverse-complement consistency penalty weighted 0.1. Early stopping used mean validation Pearson correlation with patience six. Test records were not used to choose checkpoints, architectures, hyperparameters, or variance scales.

For each ensemble, prediction was the mean of the three seed-specific means, in the spirit of bootstrap-aggregated ensemble prediction [34]. Each member s of an ensemble predicts a mean $\mu_{s}(x)$ and a learned heteroscedastic variance $\sigma_{s}^{2}(x)$. The ensemble predictive mean is $\mu (x)=\frac{1}{3}\sum_{s}\mu_{s}(x)$, and predictive uncertainty is $U_{t}(x)=\lambda_{t}\left[\frac{1}{3}\sum_{s}\sigma_{s}^{2}(x)+\frac{1}{3}\sum_{s}{\left(\mu_{s}(x)-\mu (x)\right)}^{2}\right],$ the sum of the mean within-model variance and the between-seed variance, scaled by a per-cell-type factor $\lambda_{t}$ estimated from squared prediction errors on the validation split only and then frozen (reviewer λ: 0.767, 0.768, 0.799; cross-model evaluator λ: 0.779, 0.725, 0.784 for K562, HepG2, SK-N-SH) [26,29]. The cross-model evaluation ensemble was never imported by the search code; it was applied only after candidate files and their SHA-256 hashes had been written (procedurally frozen), ensuring complete separation between generation and evaluation.

Three safeguards prevented overlap between ensemble training sequences and the sequences used for candidate generation or evaluation. First, all editing parents (600-parent evaluation, 90-parent confirmatory benchmark, 180-parent ablation subset) were drawn from the test chromosomes, whereas ensemble training used only the remaining chromosomes. Second, a near-duplicate audit covering exact identity, reverse-complement identity, primary-variant group membership, and cross-split Hamming distance up to three identified 27 cross-split pairs involving eight training-side variant groups, which were excluded before training and parent selection. Third, candidate parents were matched against deny lists containing historical identifiers, sequence hashes, and audit-flagged conflicts.

### 2.5. Editing Methods

All methods were evaluated for target cells K562, HepG2, and SK-N-SH at exact substitution budgets 1, 5, 10, and 20. Positions could be edited, at most, once along a search path.

#### 2.5.1. Random Matched Edits

Positions and non-reference alleles were sampled uniformly without replacement. The 600-parent evaluation used three independent fixed seeds (20260801–20260803). Numeric endpoints were averaged across replicates for method-level comparisons; the best random replicate was never selected. This choice keeps the comparison symmetric, because each directed method returns exactly one deterministic design per condition; the random baseline therefore estimates the expected outcome of a single uninformed draw. All three replicate outcomes are released in the candidate tables so that alternative statistics, including best-of-three, can be recomputed (Supplementary Table S16). The nine-criterion confirmatory benchmark used a single random replicate per condition, which bounds the impact of this choice given the magnitude of the observed differences.

#### 2.5.2. Greedy Malinois

At each step, every available single-nucleotide substitution was scored by the primary specificity margin, and the highest-scoring substitution was retained. No review, uncertainty, or sequence–domain constraints were enforced during search.

#### 2.5.3. Primary Beam (Compute-Matched Control)

A beam of 24 partial edit sets was expanded, one substitution at a time. Up to 96 candidates per step were retained by the primary score, which was the primary margin minus 0.05 times forward/reverse strand disagreement. Beam width, maximum expansion depth, and pre-screen size exactly matched SafeEdit-CRE. No reviewer score, uncertainty penalty, or sequence–domain reranking was used.

#### 2.5.4. SafeEdit-CRE

SafeEdit-CRE used the same primary expansion and compute budget as the primary beam. The top primary candidates were rescored by the reviewer ensemble according to

$$S(x)=M_{t}^{primary}(x)+0.3M_{t}^{reviewer}(x)-0.1D_{strand}(x)-0.05U_{reviewer}(x).$$

Candidates were prefiltered for homopolymer length above 8 and absolute parent-relative GC change above 0.12. Reranking used validation-derived, budget-specific limits for strand disagreement, reviewer uncertainty, 6-mer naturalness change, GC change, and homopolymer length. Positive primary and reviewer margins were favored; fallback scores retained a path when no candidate passed every preferred condition. A snapshot was taken independently at each requested budget.

Sequence naturalness was the position-independent mean log likelihood of all overlapping 6-mers under a background frequency model estimated from the 627,661 quality-filtered training-split sequences, with an additive pseudocount of 1.0 for every possible 6-mer. Reranking used the parent-relative change $\Delta N(x)=N(x_{edit})-N(x_{parent})$, and the acceptance threshold was the 0.05 quantile of ΔN measured on validation-set random edits, calibrated per edit budget and frozen before evaluation.

#### 2.5.5. Threshold and Weighting Rationale

All acceptance thresholds were calibrated from random edits of validation-split parents, computed once per edit budget, and frozen before any benchmark parent was evaluated (provenance/FROZEN_CONFIG.yaml, frozen 14 July 2026; relaxation factor fixed at 1.0). Strand disagreement, reviewer uncertainty, and absolute GC change used the 0.95 quantile of the validation random-edit distribution; the naturalness change used the 0.05 quantile (Supplementary Table S12). These thresholds describe the behavior of uninformed editing and were not adjusted on benchmark outcomes. The reranking weights (0.3 reviewer margin, 0.1 strand disagreement, 0.05 uncertainty) and the near-Pareto tolerances (5% relative, 0.1 absolute) were fixed design constants chosen before benchmarking; their sensitivity was established by the complete re-search ablation rather than by post-hoc analysis. Homopolymer control was layered by stage: runs longer than 8 were discarded during generation; reranking required runs of, at most, 7; and the final audit required runs of, at most, 6, so that search paths were preserved during generation while released sequences were synthesis-feasible.

### 2.6. Nine-Criterion Design Benchmark

The nine-criterion confirmatory benchmark used 90 test-split CRE parents with no overlap to development or primary-evaluation parents (six of 96 nominal parents were excluded after identifier-level overlap with an exploratory benchmark was discovered, without reference to outcomes). For each method–parent–target–budget combination, nine Boolean checks were recorded: non-negative primary target gain; positive primary margin gain; positive reviewer margin transfer computed by the pilot-stage compact CNN ensemble of the confirmatory phase, which was trained on the training partition only (Supplementary Table S13); strand disagreement within domain; reviewer uncertainty within domain; naturalness within domain; GC change within domain; homopolymer safety; and exact edit budget. Each check is defined formally in Supplementary Table S13 together with its per-budget audit thresholds, which were calibrated independently from random edits of validation parents and locked before evaluation; homopolymer safety and the exact edit budget are fixed by design. Infeasible combinations were required to remain in the result table with failed status.

The primary contrast was the paired nine-criterion pass-rate difference between SafeEdit-CRE and greedy. We used a 100,000-replicate parent-cluster bootstrap for 95% confidence intervals, preserving the dependence among the 12 target–budget conditions contributed by each parent. An exact two-sided McNemar test on pooled binary outcomes was retained as a descriptive sensitivity analysis. Stratified target–budget summaries were used to examine heterogeneity. Rejections were additionally decomposed by failing criterion and by quartile of the primary candidate margin ranked within each target–budget stratum; both analyses used only the frozen candidate table.

### 2.7. Primary Cross-Model Evaluation (600 Parents)

The primary cross-model evaluation evaluated 600 label-blind, previously unused parents with random matched, greedy, primary beam, and SafeEdit-CRE methods. Search produced 43,200 sequence rows because random editing had three replicates. For method-level statistics, the three random replicates were averaged to one numeric record per parent–target–budget combination, giving 28,800 rows. This collapsed table was used only for numeric comparisons, never to select a DNA sequence. Candidate files were frozen (SHA-256 hashes written) before cross-model-ensemble inference.

The principal endpoint was cross-model specificity-margin gain. The three secondary endpoints were, explicitly: (i) cross-model target gain; (ii) the fraction of conditions with positive cross-model margin gain; and (iii) cross-model ensemble uncertainty. For each contrast, metrics were first averaged over the 12 target–budget conditions within a parent; 100,000 bootstrap samples then resampled the 600 parents with replacement. Infeasible beam-search outcomes were retained as parent sequences with zero edits and zero gain.

### 2.8. Complete Re-Search Ablation

Seven searches were independently rerun on 180 parents: primary beam; full SafeEdit-CRE; and SafeEdit-CRE without reviewer score, without uncertainty penalty, without strand penalty, without naturalness reranking, and without the sequence prefilter. Unlike post hoc filter deletion, each ablation could generate a different sequence. The cross-model evaluation ensemble was then rerun on all 15,120 candidate rows. We verified candidate keys, exact edit budgets, finite numeric values, and separate computation of cross-model target and margin gains. Paired parent-cluster bootstrap intervals used the same procedure as the primary evaluation.

### 2.9. Candidate Tiering

The 3240 nine-criterion-benchmark candidates were organized into three computational priority tiers. Tier A required SafeEdit-CRE generation, passage of all nine criteria, non-negative target gain, positive primary and reviewer margin gains, exact edit budget, no synthesis-risk flags, and location on or near the two-objective primary–reviewer Pareto frontier. Near-Pareto candidates were within 5% relative or 0.1 absolute of the frontier. Tier B passed core activity and cross-model checks but permitted Pareto suboptimality or mild naturalness deviation. Tier C failed one or more hard checks and is retained only for diagnostic analysis. Three representative Tier A examples spanning K562, HepG2, and SK-N-SH are provided in Supplementary Table S10.

### 2.10. Software, Reproducibility, and Reporting

Preliminary development phases were run on an NVIDIA RTX 3060 Laptop GPU (NVIDIA Corporation, Santa Clara, CA, USA). Final benchmarks were run on NVIDIA RTX 3080 Ti compute nodes (NVIDIA Corporation, Santa Clara, CA, USA) under Slurm. Fixed seeds, environment records, code hashes, candidate hashes, checkpoint hashes, and run logs are provided in the permanent Zenodo archive (v1.1.0). Statistical values and figures were regenerated directly from candidate-level tables by an independent analysis script implemented with PyTorch (v2.6.0), NumPy (v2.2.5), pandas (v2.3.3), and Matplotlib (v3.10.8) [35,36,37,38]. All code, environment files, minimal reproduction scripts, core statistical inputs, candidate sequence tables, and all archived pretrained model checkpoints are included in the public release. The pilot-stage compact CNN used only for the exploratory confirmatory-phase audit was a development artifact that was not archived; the audit criteria it contributed are fixed in the released frozen candidate table, and the reproduction instructions state explicitly which stages can be reproduced end to end from the archive and which results are recomputable from the frozen candidate tables alone. Step-by-step reproduction instructions, including environment setup, archive download, integrity verification, and the stage-by-stage command path, are provided in REPRODUCTION.md in the release repository.

## 3. Results

### 3.1. Modeling the Activity Landscape for Cell-Type-Selective CRE Design

The public MPRA table contained 798,064 sequence records. After fixed quality filtering, sequence validation, length filtering, and exact-sequence deduplication, the full-data loader retained 749,054 records: 627,661 training, 58,811 validation, and 62,582 test records. Chromosomes 19, 21, and X formed validation; chromosomes 7 and 13 formed test; remaining eligible chromosomes formed training. Parent selection used identifiers and sequence hashes, never activity labels.

**Figure 2:**
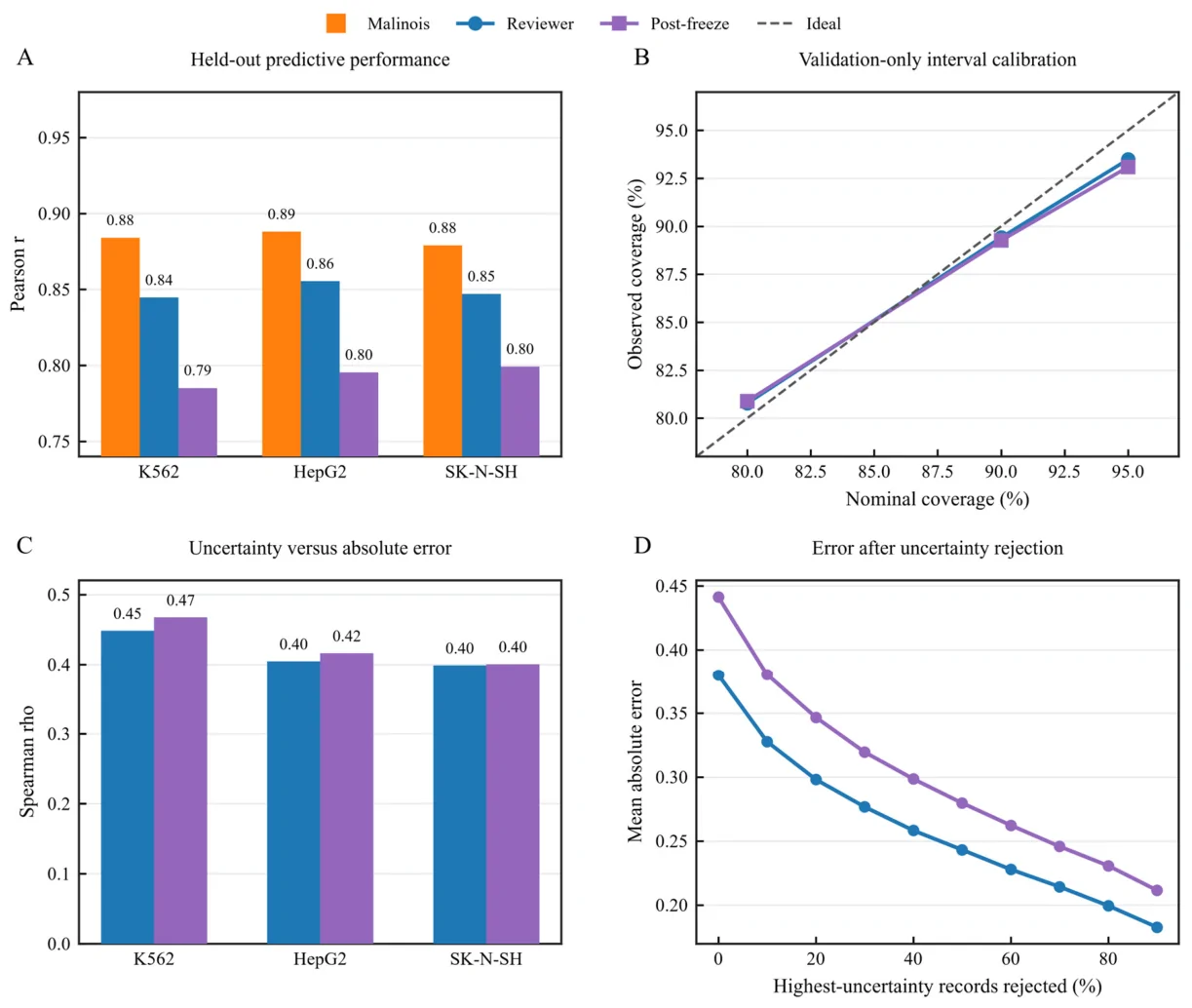
Predictive performance and uncertainty diagnostics. (**A**) Test-set Pearson correlation by cell type for the primary, reviewer, and cross-model evaluator models. (**B**) Observed test coverage at 80%, 90%, and 95% nominal levels after validation-only variance scaling; the dashed line marks ideal calibration. (**C**) Spearman association between predicted uncertainty and absolute error. (**D**) Mean test error after progressively rejecting the most uncertain records. All neural ensembles contain three independently seeded members. In panels (**A**,**C**), orange, blue, and purple bars denote the primary Malinois predictor, the reviewer ensemble, and the post-freeze cross-model evaluator, respectively; in panels (**B**,**D**), blue and purple lines denote the reviewer and post-freeze evaluator, and the dashed line in panel B marks ideal calibration.

Forward/reverse-complement averaged Malinois predictions reproduced the published test performance (mean Pearson r=0.884 and mean Spearman ρ=0.825 across the three cell types). On the same 62,582 test records, the three-member residual-dilated reviewer ensemble achieved mean Pearson r=0.849, whereas the three-member multi-seed evaluator achieved r=0.793 (Figure 2A). Their different convolutional structures allowed one ensemble to review candidates during search and the other to evaluate the frozen candidate set.

Predictive uncertainty, defined as the validation-scaled sum of mean within-model variance and between-seed variance (Methods), was formed from learned heteroscedastic variance and between-seed variation, with multiplicative scaling estimated exclusively on validation data. Across cell types, 95% test-interval coverage was 0.930–0.940 for the reviewer and 0.923–0.935 for the cross-model evaluator (Figure 2B). Reviewer uncertainty correlated with absolute error at Spearman ρ=0.399–0.448; cross-model-evaluator uncertainty gave ρ=0.400–0.467 (Figure 2C). Rejection curves showed decreasing mean absolute error as high-uncertainty records were removed (Figure 2D), supporting uncertainty-guided candidate triage.

### 3.2. SafeEdit-CRE Improves Cross-Model Cell-Type Selectivity Across 600 Held-Out Parents

We evaluated four editing methods on 600 held-out 200 nt natural CRE parents selected by SHA-256 ordering after removal of all historical parent identifiers and sequence hashes. Methods were applied for each of three target cells (K562, HepG2, SK-N-SH) and four substitution budgets (1, 5, 10, 20 nt), producing 28,800 method-level comparisons. Random editing used three independent replicates whose metrics were averaged without best-of-three selection. After candidate generation, sequences were passed to the held-out cross-model evaluator that had no role in the search.

**Figure 3:**
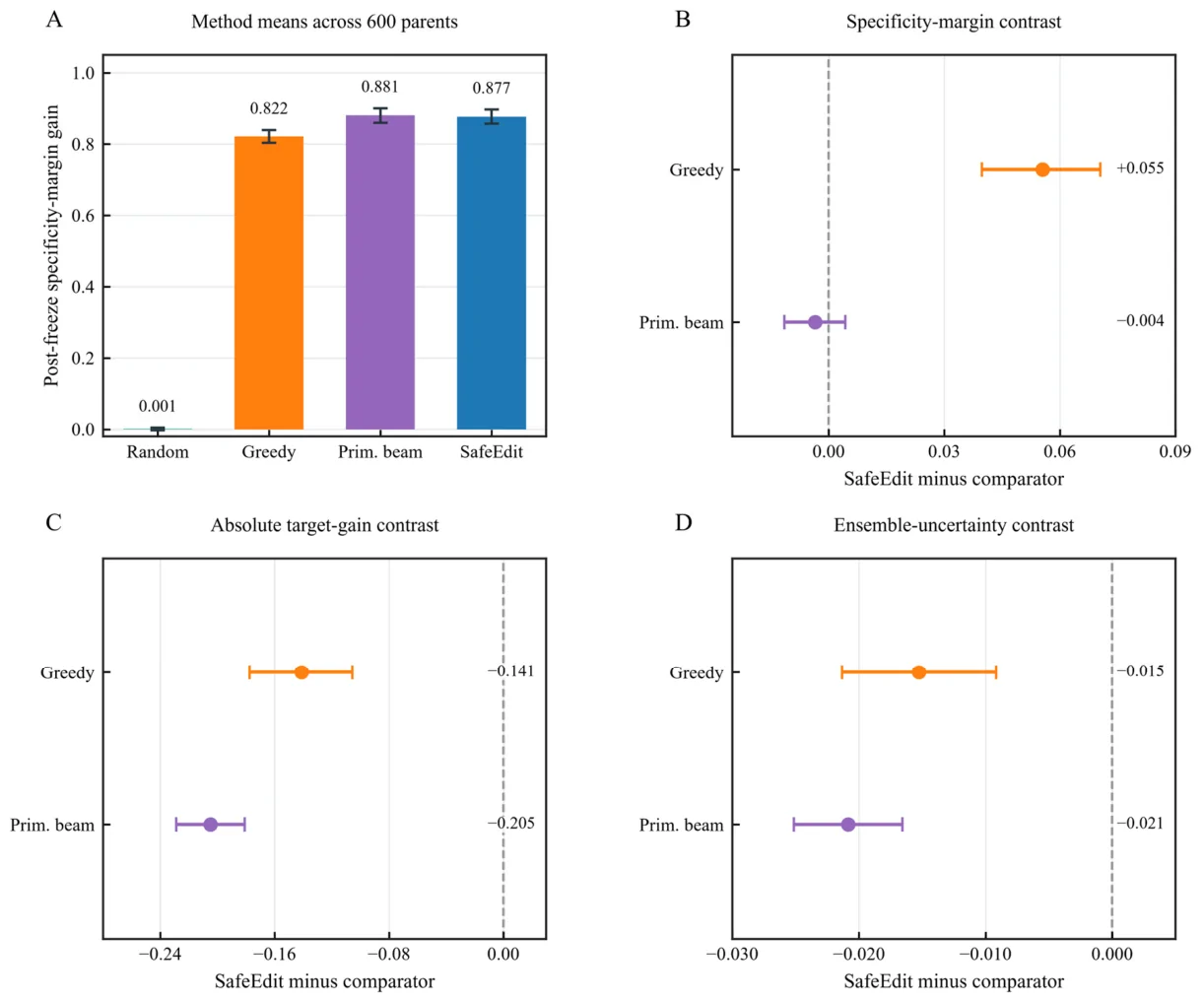
Cross-model evaluation on 600 held-out natural CRE parents. (**A**) Mean held-out cross-model specificity-margin gain by editing method. (**B**) Paired difference in margin gain for SafeEdit-CRE versus greedy and the compute-matched primary beam. (**C**) Corresponding paired difference in absolute target gain. (**D**) Corresponding paired difference in cross-model ensemble uncertainty. Points show means; intervals are 100,000-replicate parent-cluster bootstrap 95% confidence intervals. Random replicates were averaged, never selected by best outcome. Orange and purple markers denote the SafeEdit-CRE contrasts against greedy and the compute-matched primary beam, respectively; vertical dashed lines mark zero difference.

Across all 7200 parent–target–budget conditions, SafeEdit-CRE achieved mean cross-model specificity-margin gain of 0.877, compared with 0.822 for iterative greedy optimization (paired difference 0.055; parent-cluster bootstrap 95% CI, 0.040–0.071) and 0.001 for random matched editing (difference 0.876; 95% CI, 0.856–0.896) (Table 1; Figure 3A). The fraction of conditions with positive cross-model margin gain was 95.4% for SafeEdit-CRE and 97.1% for greedy, indicating that the advantage over greedy was driven by the magnitude and distribution of gains rather than by a larger fraction of positive cases.

The compute-matched primary beam search—which used the same beam width, expansion schedule, and search budget as SafeEdit-CRE but relied solely on the primary Malinois predictor for candidate ranking—achieved a mean cross-model margin gain of 0.881. SafeEdit-CRE matched this performance closely (paired difference −0.0036; 95% CI, −0.0114–0.0042; Figure 3B) while reducing absolute target gain by 0.205 (95% CI, −0.229 to −0.181) and lowering cross-model ensemble uncertainty by 0.0209 (95% CI, −0.0251 to −0.0166) (Figure 3C,D). Relative to greedy optimization, SafeEdit-CRE improved specificity margin by 0.055 (95% CI, 0.040–0.071), reduced target gain by 0.141 (95% CI, −0.177 to −0.106), and reduced uncertainty by 0.0153 (95% CI, −0.0214 to −0.0091).

The compute-matched beam shows that search breadth alone can recover the same predicted specificity. SafeEdit-CRE changes which member of that broad candidate pool is selected: its variants have lower ensemble uncertainty and smaller target-score excursions while retaining the specificity margin.

The sequence–domain prefilter returned a zero-edit outcome for 12 of 600 parents (2.0%) in at least one condition. Retaining these outcomes in all summaries preserved the prespecified denominator and quantified the practical cost of sequence safeguards.

### 3.3. Design-Criteria Benchmark on 90 Non-Overlapping Confirmatory Parents

To assess whether SafeEdit-CRE designs not only score well under cross-model evaluation but also satisfy a broad set of sequence-quality and model-agreement criteria, we applied a prespecified nine-criterion design benchmark to 90 parent sequences that did not overlap with any development or primary-evaluation parents (six nominal parents were excluded after identifier-level overlap with an exploratory benchmark was discovered). For each method–parent–target–budget combination, we recorded non-negative primary target gain, positive primary margin gain, positive reviewer margin transfer, strand disagreement within domain, reviewer uncertainty within domain, naturalness within domain, GC change within domain, homopolymer safety, and exact edit budget. Thresholds were derived from validation-set random edits and locked before evaluation.

**Figure 4:**
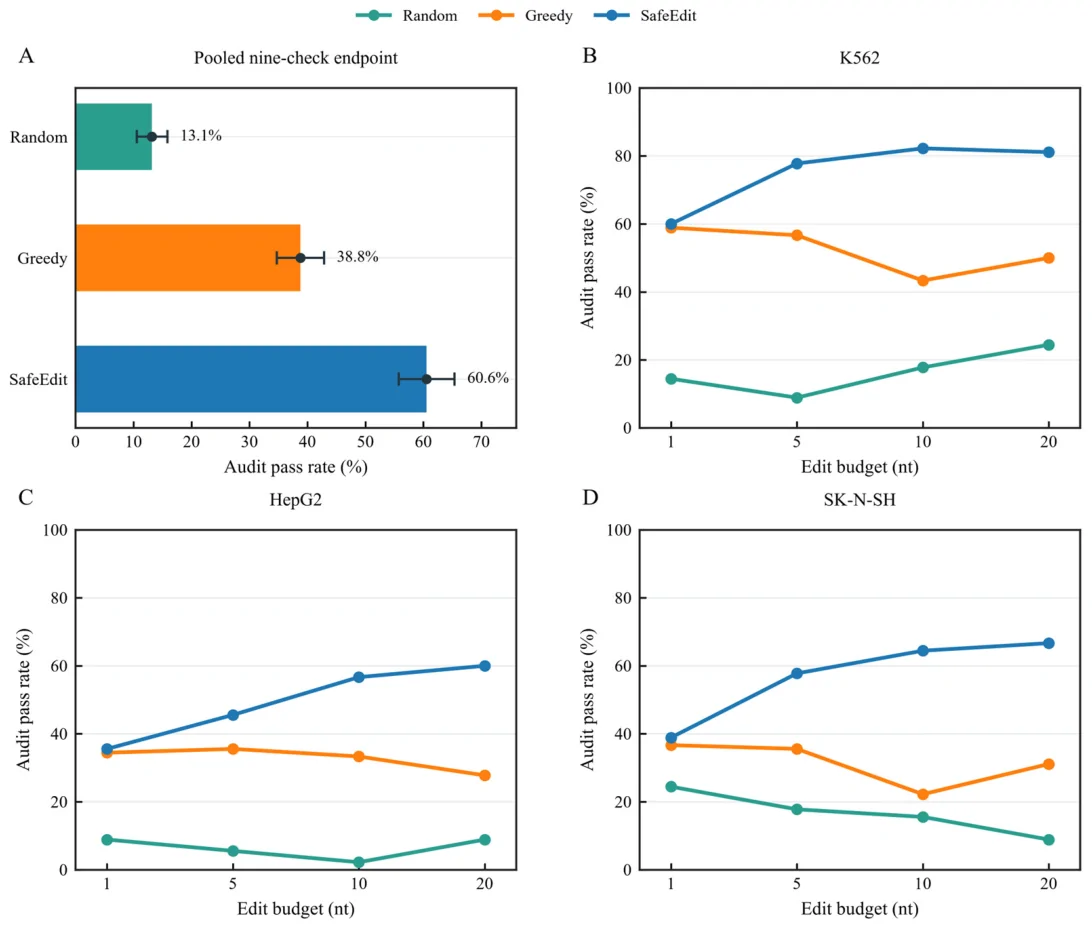
Nine-criterion design benchmark. (**A**) Pooled nine-criterion pass rates in the locked non-overlapping 90-parent confirmatory benchmark. Error bars are parent-cluster bootstrap 95% confidence intervals. (**B**–**D**) Pass rates stratified by edit budget for K562, HepG2, and SK-N-SH, respectively. Teal, orange, and blue bars/lines denote random matched editing, greedy Malinois, and SafeEdit-CRE, respectively.

SafeEdit-CRE passed all nine criteria for 654 of 1080 designs (60.6%), compared with 419 of 1080 greedy designs (38.8%) and 142 of 1080 random designs (13.1%) (Table 2; Figure 4A). The paired SafeEdit–greedy difference was 21.8 percentage points (parent-cluster bootstrap 95% CI, 18.3–25.3 percentage points). The SafeEdit–random difference was 47.4 percentage points (95% CI, 42.5–52.2 percentage points). The advantage over greedy was small at a one-base budget and increased at budgets 5–20 (Figure 4B–D).

The 21.8 percentage-point difference reflects the combined effect of reviewer, uncertainty, and sequence–domain criteria during candidate generation. Post hoc screening of greedy designs recovered markedly fewer variants that met the same prespecified criteria.

To characterize which designs fail and why, we decomposed rejections by failing criterion (Supplementary Table S14; Supplementary Figure S4A). For SafeEdit-CRE, 426 of 1080 designs failed at least one criterion. The dominant failure was absence of reviewer margin transfer, accounting for 302 failures, of which 252 were the only failing criterion; homopolymer safety accounted for 103 failures, 64 as the only cause, with its failure rate declining from 11.9% at one edit to 7.0% at twenty edits. Sequence–domain and uncertainty gates fired infrequently: strand disagreement 13, reviewer uncertainty 10, naturalness 11, and GC change 7, and no confirmatory design was infeasible. Greedy designs failed most often on reviewer transfer, with 352 failures, and on strand disagreement, with 221; random designs failed predominantly on basic activity criteria: target gain 535, reviewer transfer 540, and primary margin 509. The corresponding generation-stage effects are quantified by the re-search ablation: removing the sequence prefilter eliminated all 48 infeasible outcomes, and removing naturalness reranking changed margin gain from 0.890 to 0.979 and uncertainty from 0.312 to 0.343.

In a descriptive post-freeze analysis of the selected designs—not of within-search candidate ranks, which were not persisted—acceptance rates across quartiles of the primary candidate margin were 64.1%, 58.7%, 61.7%, and 57.6% for SafeEdit-CRE, compared with a monotonic increase from 7.2% to 18.5% for random editing (Supplementary Table S15; Supplementary Figure S4B). No monotonic association was apparent in these aggregated rates. This pattern is consistent with, but does not establish, a greater role for cross-model and sequence–domain criteria relative to primary-score magnitude in determining which SafeEdit-CRE designs are retained.

### 3.4. Cell-Type and Edit-Budget Effects

**Figure 5:**
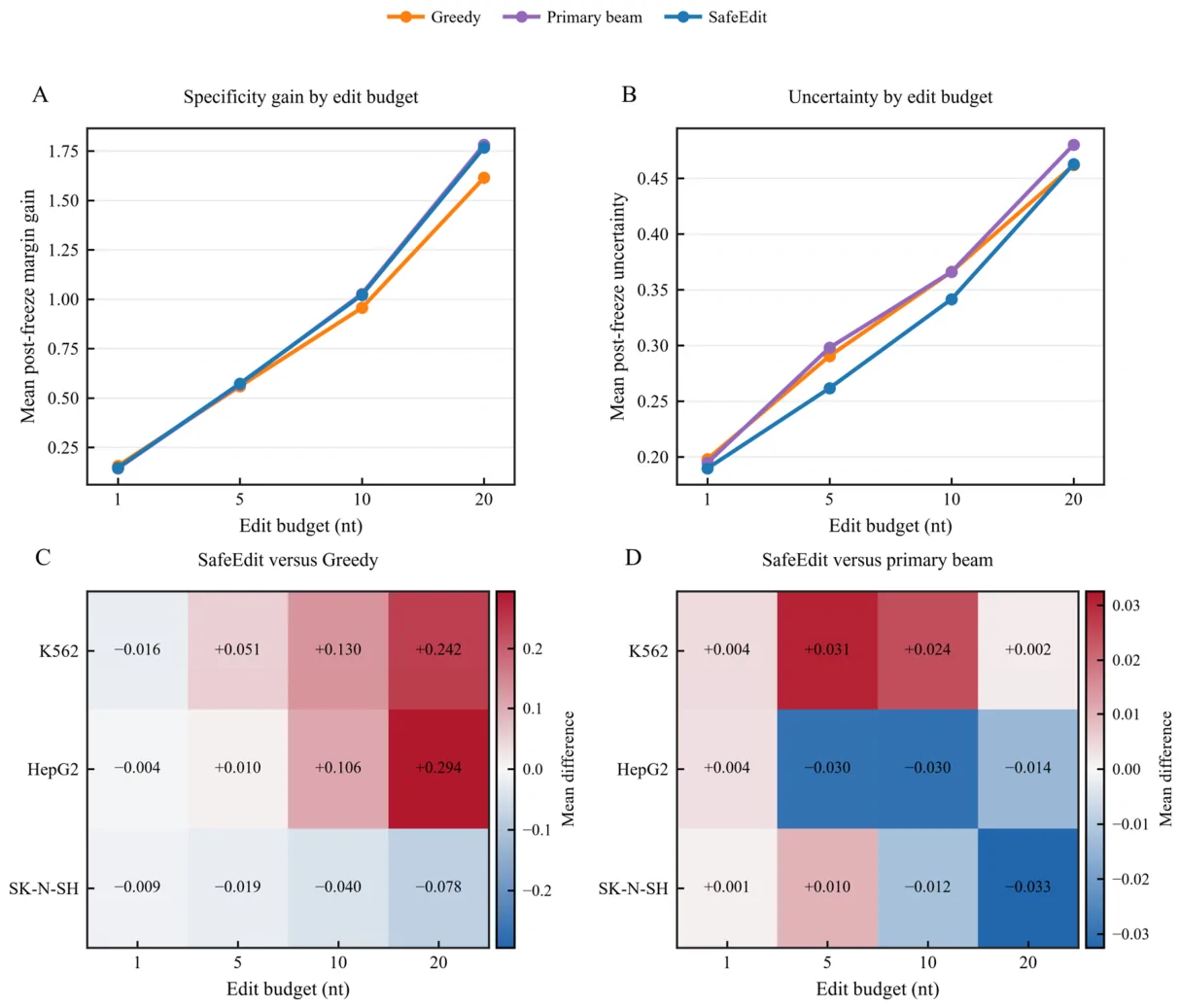
Cell-type and edit-budget effects. (**A**,**B**) Cross-model specificity-margin gain and uncertainty pooled over the three target cells across edit budgets for greedy, primary-beam, and SafeEdit-CRE searches. (**C**,**D**) Target-by-budget differences in mean specificity-margin gain for SafeEdit-CRE relative to greedy or the compute-matched primary beam. Random editing is omitted from panels (**A**,**B**) because its pooled margin gain remains near zero. Lines and heatmap cells are descriptive means over parents. Edit budgets are reported in nt (nucleotide substitutions). Orange, purple, and blue lines denote greedy, primary-beam, and SafeEdit-CRE searches, respectively; in panels (**C**,**D**), red and blue heatmap cells denote positive and negative mean differences, respectively.

The benefit of cross-model selection varied with target cell type and edit budget (Figure 5). For K562, the SafeEdit–greedy cross-model margin difference progressed from −0.016 at one edit to 0.242 at 20 edits; for HepG2, it progressed from −0.004 to 0.294. In SK-N-SH, differences ranged from −0.078 to −0.033 across budgets, indicating cell-type-specific heterogeneity in the activity landscape.

Against the compute-matched primary beam, SafeEdit-CRE differences remained within tight equivalence bounds in all strata: K562 0.004–0.031; HepG2 −0.030–0.004; SK-N-SH −0.033–0.010, consistent with the pooled comparison across target cells and edit budgets.

The pattern is consistent with consensus reranking becoming increasingly valuable as the number of plausible edit paths grows. At one substitution, all three directed strategies are frequently constrained to the same small neighborhood of the parent sequence; at 5–20 substitutions, cross-model review filters out edits that look favorable under the primary predictor but transfer poorly to the held-out ensembles. The SK-N-SH result suggests that design thresholds should be calibrated per cell type rather than transferred unchanged across cellular models.

### 3.5. Complete Re-Search Ablation Identifies Key Components

To identify which SafeEdit-CRE components drive the observed behavior, we reran the complete search under seven configurations on 180 parents (15,120 rows). Each configuration generated its own candidate sequences; filters were not removed retrospectively from a shared candidate table. Cross-model inference was recomputed for every candidate after each search.

**Figure 6:**
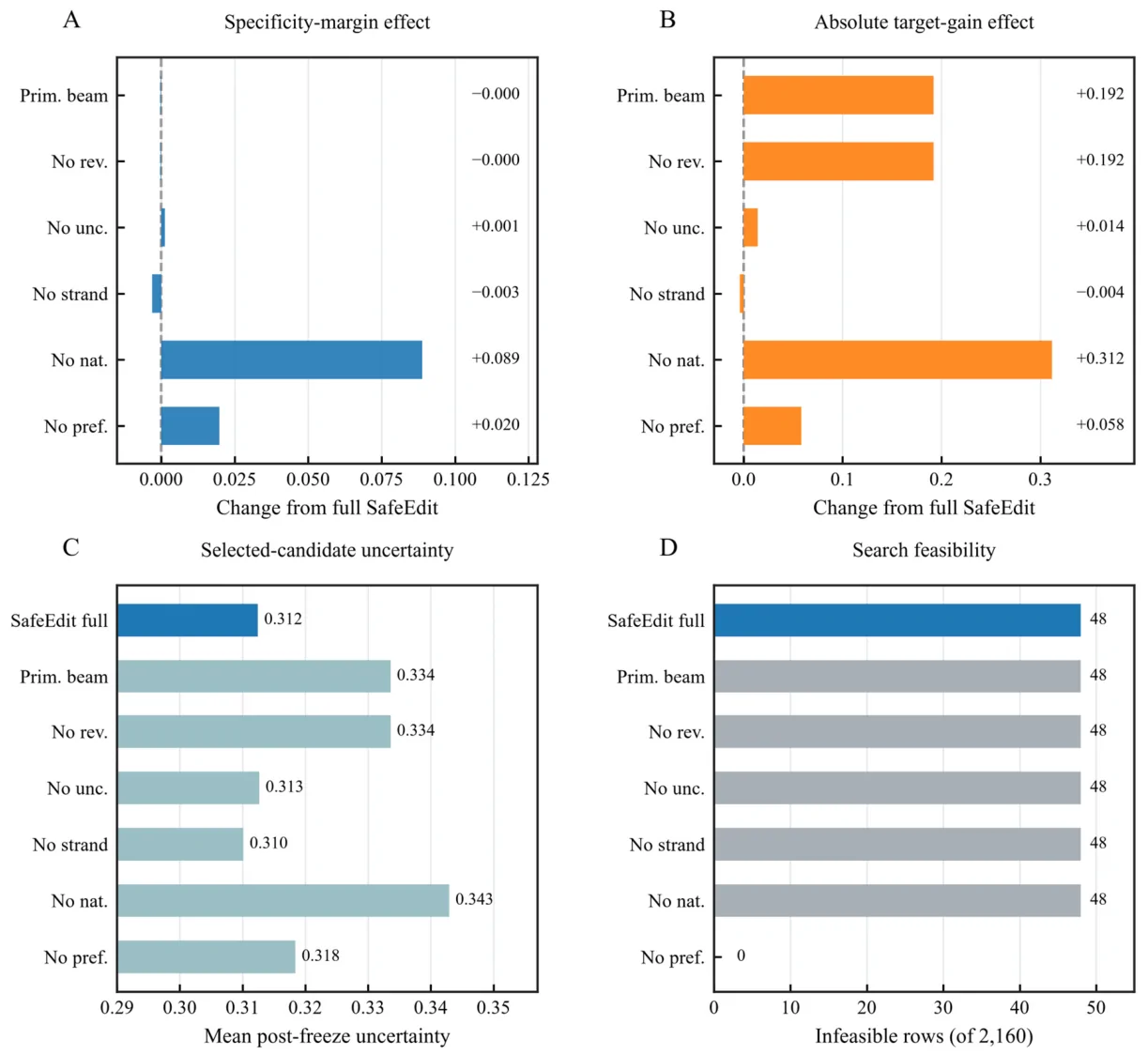
Complete re-search ablation on 180 parents. (**A**) Change in cross-model specificity-margin gain relative to full SafeEdit-CRE. (**B**) Corresponding change in absolute target gain. (**C**) Mean cross-model ensemble uncertainty for selected candidates. (**D**) Number of infeasible search rows. Each configuration was independently rerun. Naturalness-aware reranking is the primary driver of uncertainty reduction, while prefilter enforcement trades margin for synthesis feasibility. In panel (**A**), blue bars show changes in specificity-margin gain relative to full SafeEdit-CRE; in panel (**B**), orange bars show changes in absolute target gain. In panels (**C**,**D**), dark-blue bars denote full SafeEdit-CRE, whereas light-teal and gray bars denote the ablation configurations; vertical dashed lines in panels (**A**,**B**) mark zero change.

Full SafeEdit-CRE yielded mean cross-model margin gain 0.890, target gain 1.705, and uncertainty 0.312 (Figure 6). The matched primary beam had margin gain 0.890 (paired full-minus-primary difference 0.0002; 95% CI, −0.0145–0.0151), target gain 1.897 (difference −0.192; 95% CI, −0.244 to −0.143), and uncertainty 0.334 (difference −0.0212; 95% CI, −0.0283 to −0.0140).

Removing naturalness reranking raised cross-model margin gain to 0.979 and target gain to 2.017, together with an uncertainty increase to 0.343. Full SafeEdit-CRE reduced uncertainty by 0.0305 (95% CI, 0.0218–0.0394) relative to this configuration while moderating margin by 0.088 (95% CI, 0.075–0.102) and target gain by 0.312 (95% CI, 0.266–0.360). Removing the sequence–domain prefilter eliminated all 48 infeasible rows and raised margin gain to 0.910 by relaxing the GC and homopolymer limits. Strand and uncertainty penalties had smaller pooled effects, whereas the reviewer-free configuration reproduced the primary beam exactly.

Naturalness-aware reranking made the largest contribution to uncertainty reduction, whereas the sequence–domain prefilter determined synthesis feasibility. Removing the reviewer reproduced the primary-beam result exactly, showing that the reviewer changes the returned sequence rather than merely annotating it after selection.

### 3.6. Tier A Candidate Library and Representative Examples

From the 3240 designs in the 90-parent confirmatory benchmark, we derived a tiered candidate library for experimental screening. Seventy-four Tier A sequences were generated by SafeEdit-CRE, passed all nine criteria, met target and margin requirements, had no synthesis-risk flag, and lay on or near the primary–reviewer Pareto frontier (Supplementary Table S9; Supplementary Figure S3). Tier B contained 1141 alternatives that passed core activity and cross-model checks but were Pareto-dominated or mildly outside a sequence-quality criterion; the remaining 2025 Tier C sequences were retained for failure-mode analysis. The resulting 74-sequence Tier A panel spans all three target cell types and four edit budgets, providing a compact first-pass reporter-screening set, while Tier B retains sufficient breadth for a larger secondary MPRA library.

To illustrate the nature of SafeEdit-CRE designs, Supplementary Table S10 presents three representative Tier A candidates—one each for K562, HepG2, and SK-N-SH—all generated under a five-edit budget and achieving Pareto-frontier status without synthesis risk flags. Each example reports the parent and edited 200 nt sequence, edit positions, primary and reviewer specificity-margin gains, primary target-cell activity gain, the reduction in cross-model predictive uncertainty, GC content, naturalness, and homopolymer status. All three show positive primary target gain and positive reviewer margin transfer with decreased cross-model uncertainty. Local sequence changes near edited positions are recorded as testable sequence-grammar hypotheses for experimental follow-up.

## 4. Discussion

SafeEdit-CRE was designed around a practical regulatory-genomics question: how can a natural cis-regulatory element be changed with a small number of substitutions while retaining a cell-type-selective signal when the selected sequence is inspected by a second model? Existing sequence-to-activity workflows provide strong predictors and increasingly capable design tools, but candidate ranking is often tied to the same predictor used as the optimization objective [24,25,27]. SafeEdit-CRE separates those roles and uses cross-model selection to decide which minimal edits are worth carrying forward.

The clearest separation from iterative greedy optimization appeared in the 600-parent evaluation. SafeEdit-CRE increased cross-model specificity-margin gain by 0.055 margin units (95% CI 0.040–0.071), even though greedy already produced a positive margin gain in 97.1% of conditions. The difference is, therefore, a ranking effect among mostly plausible edits: the reviewer and uncertainty criteria favor variants with larger gains that remain consistent across models. The effect was largest at 5–20 nucleotide budgets in K562 and HepG2, where more edit paths are available and primary-model preferences can diverge.

Search breadth alone did not account for this behavior. The compute-matched primary beam achieved a margin gain of 0.881, closely matching SafeEdit-CRE’s 0.877 (difference −0.004; 95% CI −0.011–0.004). SafeEdit-CRE nevertheless returned variants with lower cross-model uncertainty (0.314 versus 0.335) and smaller target-score excursions. In other words, the framework retains the predicted cell-type-specific objective while choosing a less dispersed part of the candidate landscape. That trade-off is especially useful when synthesis and reporter capacity are limited and the experimental budget is better spent on a compact, auditable panel.

The nine-criterion benchmark gives the same result in a more demanding design setting. SafeEdit-CRE passed 60.6% of designs, compared with 38.8% for greedy optimization, for a 21.8 percentage-point difference (95% CI 18.3–25.3). The complete re-search ablation shows why: naturalness-aware reranking makes the largest contribution to uncertainty reduction, while the sequence–domain prefilter enforces GC and homopolymer feasibility during generation. Removing the reviewer reproduced the primary-beam result exactly, identifying cross-model review as the component that changes which sequence is returned rather than merely annotating it afterward.

The output is consequently a candidate library rather than a single model optimum. The 74 Tier A sequences passed all nine criteria, met the target and margin requirements, carried no synthesis-risk flag, and lay on or near the primary–reviewer Pareto frontier. They span three target cell types and four edit budgets, providing a compact, computationally prioritized first-pass panel for reporter screening and downstream regulatory-grammar dissection; 1141 Tier B sequences retain breadth for a larger follow-up MPRA. Candidate records preserve the parent sequence, edited sequence, edit positions, model-specific gains, uncertainty, strand consistency, naturalness, base composition, homopolymer status, and edit count. The three representative examples in Supplementary Table S10 highlight concrete sequence-grammar hypotheses: localized substitutions that shift cell-type selectivity while preserving the broader CRE scaffold, directly testable via reporter assays.

Several design choices make the comparison interpretable. Parent selection was label-blind, development and evaluation parents were separated by identifiers and sequence hashes, random replicates were averaged rather than selected by outcome, and confidence intervals resampled whole parents. The post-freeze evaluator was excluded from candidate generation, while the compute-matched beam kept search width and expansion cost fixed. The complete re-search ablation further ensured that component effects reflect different returned sequences instead of retrospective filtering of one candidate table.

The immediate computational deliverable is a compact, auditable set of CRE variants for reporter screening. The present results establish the computational ranking and its cross-model behavior; reporter assays can now test whether the predicted margin and uncertainty ordering track measured activity. Because the candidates retain natural CRE scaffolds and explicit edit budgets, the panel is also suitable for follow-up experiments that map local sequence changes to cell-type-selective regulatory output. Beyond reporter assays, the minimal-editing framework may inform the design of cell-type-specific regulatory elements and motivate follow-up studies using base or prime editing: the Tier A panel can guide the construction of synthetic gene circuits, help prioritize functional noncoding variants for experimental validation in disease cohorts, and provide a template for rationally modifying endogenous CREs. Each candidate comes with a documented edit budget, strand-consistency checks, and naturalness scores, which may help reduce the experimental risk of pursuing sequences that fail synthesis or produce uninterpretable results.

SafeEdit-CRE thus provides a reproducible route from sequence-to-activity prediction to experimentally testable minimal edits. Cross-model selection preserves the primary cell-type-specific objective, reduces predictive dispersion relative to a compute-matched single-model search, and produces a practical Tier A panel instead of an opaque list of unconstrained high-scoring sequences. The same selection principle can be transferred to other regulatory-prediction models and cellular contexts where candidate reliability matters as much as the nominal optimum.

## 5. Conclusions

SafeEdit-CRE establishes cross-model uncertainty-aware minimal editing as a practical route for converting sequence-to-activity models into compact, cell-type-selective CRE variant libraries. By retaining natural sequence scaffolds and explicitly controlling edit distance, model agreement, and predictive uncertainty, the framework provides a focused starting point for functional-genomics studies of regulatory sequence grammar.

## Figures and Tables

**Table 1:** Cross-model evaluation outcomes across 600 held-out parents.

Method	Mean Margin Gain	Mean Target Gain	Positive Margin (%)	Mean Uncertainty
Random matched	0.001	0.012	48.6	0.185
Greedy Malinois	0.822	1.803	97.1	0.329
Primary beam (compute-matched)	0.881	1.866	94.8	0.335
SafeEdit-CRE	0.877	1.661	95.4	0.314

**Table 2:** Nine-criterion design benchmark across 90 non-overlapping confirmatory parents.

Method	Designs	Passed	Pass Rate (%)	Parent-Bootstrap 95% CI (%)
Random matched	1080	142	13.1	11.1–15.1
Greedy Malinois	1080	419	38.8	35.7–42.0
SafeEdit-CRE	1080	654	60.6	56.9–64.3

## Data Availability

The processed MPRA measurements used to train Malinois are provided in Supplementary Table S2 of Gosai et al. [21]. The associated raw data, processing notebooks, model weights, and immunofluorescence images are archived at Zenodo record 10698014. The code, frozen evaluation outputs, and candidate library used in this study are publicly available in SafeEdit-CRE v1.1.0 at GitHub (commit 3140e7115ced9a3d25e8c9d52e81df3be416d7c8, https://github.com/Haangyeon/SafeEdit-CRE/releases/tag/v1.1.0, accessed on 26 August 2026) and Zenodo (version DOI: https://doi.org/10.5281/zenodo.21478233; concept DOI: https://doi.org/10.5281/zenodo.21458488). The repository additionally includes a minimal end-to-end reproduction script that regenerates the main figures and statistical tables from the released summary inputs. Pretrained model checkpoints are included in the Zenodo archive.

## Supplementary Materials

The following supporting information can be downloaded at: https://www.mdpi.com/article/10.3390/genes17091062/s1, Table S1: Quality-filtered full-data partitions used for G7 model training; Table S2: Ensemble performance on the 62,582-record test split; Table S3: Validation-scaled interval coverage on the test split; Table S4: G4 audit pass rates by target cell and edit budget; Table S5: Pooled G8 method summaries; Table S6: Pooled paired G8 contrasts; Table S7: G9 post-freeze outcomes for seven independently rerun search configurations; Table S8: Selected G9 paired contrasts; Table S9: Frozen G4 candidate tiers; Table S10: Three representative Tier A reporter-screening candidates; Table S11: Training details and seeds for the reviewer and cross-model evaluation ensembles; Table S12: Documentation of all parameters and thresholds; Table S13: Formal definitions of the nine Boolean design-quality checks and audit thresholds; Table S14: Nine-criterion rejection decomposition in the 90-parent confirmatory benchmark; Table S15: Nine-criterion acceptance rate by quartile of the primary candidate-margin rank; Table S16: Best-of-three sensitivity analysis for random matched editing; Figure S1: Uncertainty rejection and interval calibration; Figure S2: Target-budget heterogeneity in the G8 post-freeze endpoint; Figure S3: Computational candidate tiers; Figure S4: Rejection decomposition and primary-rank acceptance.
