# Supplementary material for: Cross-Model Uncertainty-Aware Minimal Editing of Cis-Regulatory Elements for Cell-Type-Selective Design
Source: Genes (Basel). 2026 Sep 1;17(9):1062. doi: 10.3390/genes17091062 (PMC13606248; doi:10.3390/genes17091062)
Supplement: Supplementary file 1 [file genes-17-01062-s001.zip › genes-4487654-supplementary.pdf]

Supplementary Information

Cross-model uncertainty-aware minimal editing of cis-regulatory elements for cell-type-selective design

Angran Xia[1], Changwei Wang[1,*], Yemao Xia[2]

[1]School of Chemistry and Chemical Engineering, Shaanxi Normal University,
620 West Chang'an Avenue, Chang'an District, Xi'an, Shaanxi 710119, China
[2]College of Science, Nanjing Forestry University,
159 Longpan Road, Xuanwu District, Nanjing, Jiangsu 210037, China

Correspondence: `haangyeon@snnu.edu.cn` (A.X.); `snnu.changweiwang@snnu.edu.cn` (C.W.)

Contents

1 Supplementary methods and audit notes **2**
- 1.1 Study phases and separation of roles . . . 2
- 1.2 Integrity checks . . . 2

2 Supplementary results **2**
- 2.1 Data partitions and predictor performance . . . 2
- 2.2 G4 hard-audit stratification . . . 2
- 2.3 G8 post-freeze endpoint and heterogeneity . . . 4
- 2.4 G9 true-search ablation . . . 5
- 2.5 Candidate library . . . 5
- 2.6 Representative Tier A candidates . . . 7

3 Supplementary methods and thresholds **9**
- 3.1 Ensemble training details, thresholds, and criteria definitions . . . 9
- 3.2 Rejection decomposition and primary-rank acceptance . . . 10

4 Reproducibility summary **12**

1 Supplementary methods and audit notes

1.1 Study phases and separation of roles

The study was executed in sequential phases. G0–G3 established the data audit, published-model reproduction, pilot CNN reviewer, and initial editing workflow. G4 froze a 90-parent, three-method hard-audit benchmark (six nominal parents excluded for overlap with the G4 exploratory pilot). G6 assigned computational candidate tiers. G7 trained the full-data residual-dilated reviewer and the multi-seed post-freeze evaluator. G8 selected 600 new parents and compared four search methods. G9 reran seven complete search configurations on 180 parents. G10 applied the post-freeze endpoint and assembled summary analyses. Activity labels were never used to select parents or candidate sequences.

The terms *reviewer* and *post-freeze evaluator* denote different roles. The reviewer was available to SafeEdit during G8/G9 search. The post-freeze evaluator was loaded only after candidate files were frozen. Both were trained on the same public MPRA source table; hence “post-freeze” describes procedural separation from search, not external biological independence.

1.2 Integrity checks

The final G9 table contains 15,120 rows, 180 unique parents, seven configurations, three target cells, and four budgets. There are no duplicate parent–target–budget–configuration keys, no non-finite numeric values, and no exact edit-budget violations among feasible designs. The G8 collapsed table contains 28,800 method-level rows and 600 parents. Random metrics are means of three independent replicates; the collapsed table must not be used to select a particular DNA sequence.

All submission payloads were rechecked after manuscript generation. The release manifest contains SHA-256 checksums for every payload file and excludes the manifest itself from self-hashing.

2 Supplementary results

2.1 Data partitions and predictor performance

Table S1. Quality-filtered full-data partitions used for G7 model training.

Partition	Records	Chromosomes	Use
Training	627,661	Eligible chromosomes excluding validation/test	Parameter fitting
Validation	58,811	19, 21, X	Checkpoint choice and variance scaling
Test	62,582	7, 13	Final predictive diagnostics
Total	749,054	–	After quality filtering and exact deduplication

Table S2. Ensemble performance on the 62,582-record test split, recomputed from saved candidate-independent prediction tables.

Model	Cell	Pearson r	Spearman ρ	MAE	Uncertainty–error ρ
Reviewer	K562	0.845	0.781	0.396	0.448
Reviewer	HepG2	0.855	0.809	0.354	0.404
Reviewer	SK-N-SH	0.847	0.810	0.390	0.399
Post-freeze evaluator	K562	0.785	0.719	0.463	0.467
Post-freeze evaluator	HepG2	0.795	0.743	0.417	0.416
Post-freeze evaluator	SK-N-SH	0.799	0.760	0.444	0.400

2.2 G4 hard-audit stratification

Table S3. Validation-scaled interval coverage on the test split.

Model	Cell	80% interval	90% interval	95% interval
Reviewer	K562	0.815	0.897	0.935
Reviewer	HepG2	0.794	0.885	0.930
Reviewer	SK-N-SH	0.814	0.900	0.940
Post-freeze evaluator	K562	0.821	0.900	0.935
Post-freeze evaluator	HepG2	0.789	0.879	0.923
Post-freeze evaluator	SK-N-SH	0.816	0.899	0.935

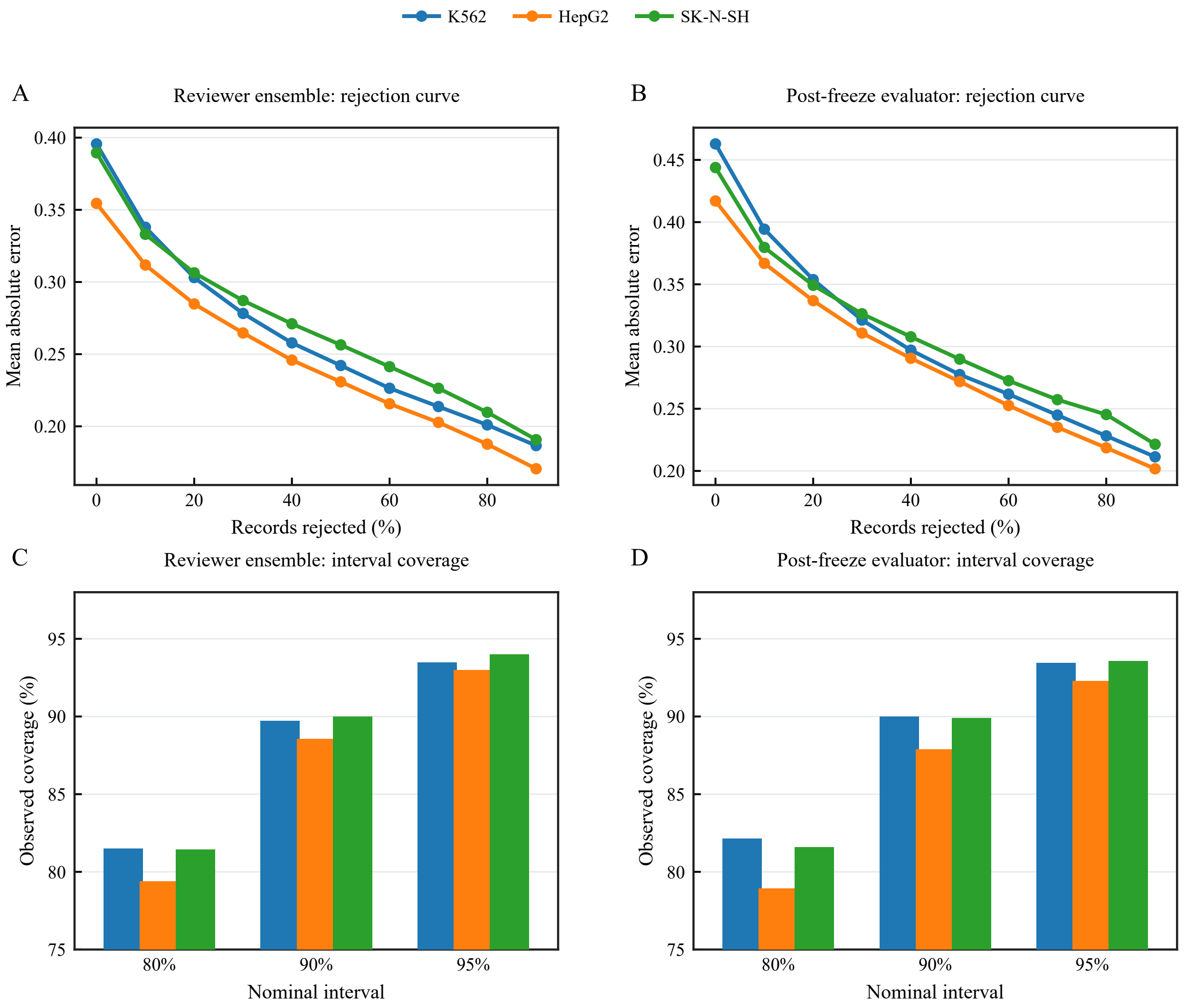

Figure S1. Uncertainty rejection and interval calibration. A–B, mean absolute error for the reviewer and post-freeze ensembles as records with the largest predicted uncertainty are removed from the held-out test set. **C–D**, observed test coverage at 80%, 90%, and 95% nominal levels by cell type. Curves and bars are descriptive test diagnostics; variance scales were fixed from validation only.

Table S4. G4 audit pass rates by target cell and edit budget. Each row contains 90 non-overlapping parents per method after excluding six parents that overlapped the G4 exploratory pilot.

Target	Budget	Random (%)	Greedy (%)	SafeEdit (%)	SafeEdit–Greedy (pp)
K562	1	14.4	58.9	60.0	1.1
K562	5	8.9	56.7	77.8	21.1
K562	10	17.8	43.3	82.2	38.9
K562	20	24.4	50.0	81.1	31.1

Target	Budget	Random (%)	Greedy (%)	SafeEdit (%)	SafeEdit–Greedy (pp)
HepG2	1	8.9	34.4	35.6	1.1
HepG2	5	5.6	35.6	45.6	10.0
HepG2	10	2.2	33.3	56.7	23.3
HepG2	20	8.9	27.8	60.0	32.2
SK-N-SH	1	24.4	36.7	38.9	2.2
SK-N-SH	5	17.8	35.6	57.8	22.2
SK-N-SH	10	15.6	22.2	64.4	42.2
SK-N-SH	20	8.9	31.1	66.7	35.6

2.3 G8 post-freeze endpoint and heterogeneity

Table S5. Pooled G8 method summaries. Each method has 7,200 parent–target–budget records after averaging the three random replicates.

Method	Margin gain	Target gain	Positive margin (%)	Uncertainty
Random matched	0.001	0.012	48.6	0.185
Greedy Malinois	0.822	1.803	97.1	0.329
Primary beam	0.881	1.866	94.8	0.335
SafeEdit consensus	0.877	1.661	95.4	0.314

Table S6. Pooled paired G8 contrasts. Differences are SafeEdit minus comparator; confidence intervals use 100,000 parent-cluster bootstrap replicates.

Comparator	Metric	Mean difference	95% CI
Greedy	Post-freeze margin gain	0.0555	0.0398 to 0.0705
Greedy	Post-freeze target gain	-0.1412	-0.1774 to -0.1057
Greedy	Post-freeze uncertainty	-0.0153	-0.0214 to -0.0091
Primary beam	Post-freeze margin gain	-0.0036	-0.0114 to 0.0042
Primary beam	Post-freeze target gain	-0.2048	-0.2294 to -0.1808
Primary beam	Post-freeze uncertainty	-0.0209	-0.0251 to -0.0166
Random matched	Post-freeze margin gain	0.8763	0.8562 to 0.8960
Random matched	Post-freeze target gain	1.6494	1.6034 to 1.6950
Random matched	Post-freeze uncertainty	0.1285	0.1222 to 0.1348

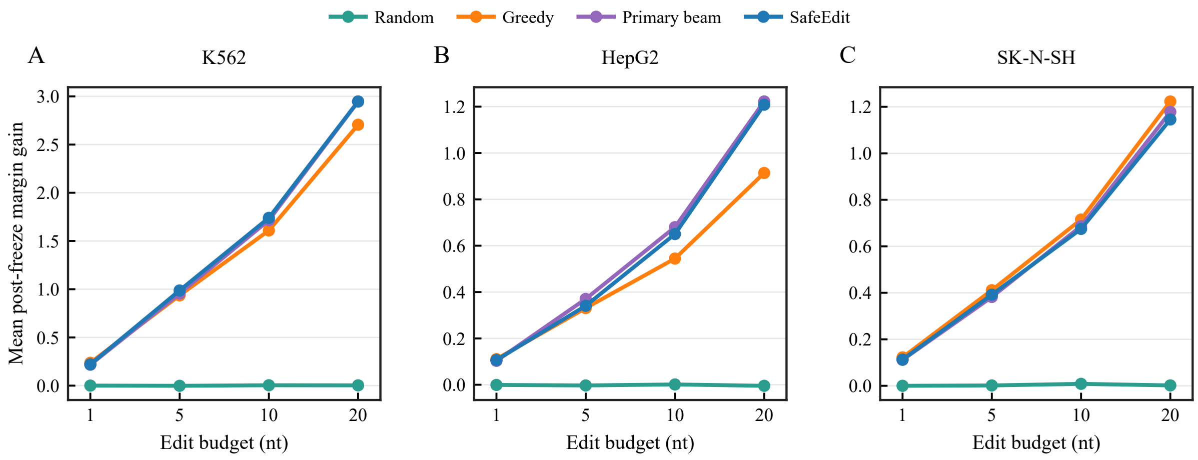

Figure S2. Target–budget heterogeneity in the G8 post-freeze endpoint. A–C, mean post-freeze specificity-margin gain across edit budgets for random, greedy, primary-beam, and SafeEdit searches in K562, HepG2, and SK-N-SH. Each point summarizes 600 parents. Values are descriptive and are not multiplicity-adjusted.

2.4 G9 true-search ablation

Table S7. G9 post-freeze outcomes for seven independently rerun search configurations. Each configuration contains 2,160 rows from 180 parents, three targets, and four budgets.

Configuration	Margin gain	Target gain	Positive (%)	Uncertainty	Infeasible
Primary beam	0.890	1.897	94.7	0.334	48
SafeEdit full	0.890	1.705	95.3	0.312	48
No reviewer score	0.890	1.897	94.7	0.334	48
No uncertainty penalty	0.891	1.719	95.2	0.313	48
No strand penalty	0.887	1.701	95.4	0.310	48
No naturalness rerank	0.979	2.017	95.3	0.343	48
No sequence prefilter	0.910	1.763	98.1	0.318	0

Table S8. Selected G9 paired contrasts. Differences are full SafeEdit minus the ablation; intervals use 100,000 parent-cluster bootstrap replicates.

Comparator	Metric	Mean difference	95% CI
Primary beam	Post-freeze margin gain	0.0002	-0.0145 to 0.0151
Primary beam	Post-freeze target gain	-0.1920	-0.2440 to -0.1432
Primary beam	Post-freeze uncertainty	-0.0212	-0.0283 to -0.0140
No naturalness	Post-freeze margin gain	-0.0888	-0.1022 to -0.0754
No naturalness	Post-freeze target gain	-0.3117	-0.3600 to -0.2658
No naturalness	Post-freeze uncertainty	-0.0305	-0.0394 to -0.0218
No prefilter	Post-freeze margin gain	-0.0198	-0.0387 to -0.0043
No prefilter	Post-freeze target gain	-0.0581	-0.1021 to -0.0227
No prefilter	Post-freeze uncertainty	-0.0060	-0.0102 to -0.0024

2.5 Candidate library

Table S9. Frozen G4 candidate tiers.

Tier	Candidates	Fraction (%)	Intended use
A	74	2.3	Highest-priority experimental panel
B	1,141	35.2	Alternative computational candidates
C	2,025	62.5	Method and failure-mode analysis

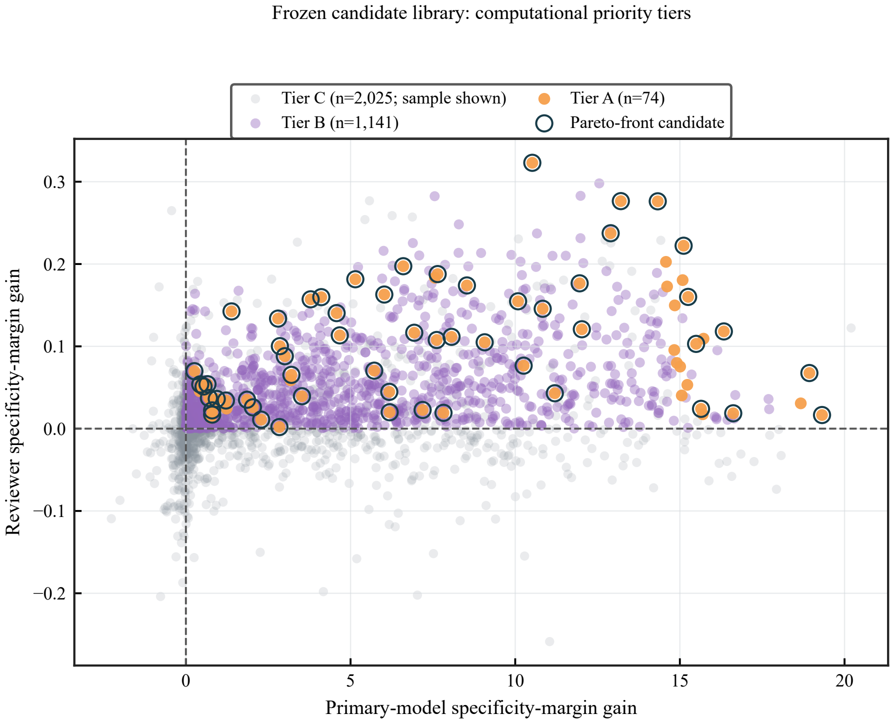

Figure S3. Computational candidate tiers. Primary-model and reviewer specificity-margin gains are shown for the frozen 3,240-sequence G4 library. All Tier A and Tier B candidates are plotted; Tier C is downsampled for legibility. Outlined points lie on the computed Pareto frontier. Tier labels represent computational priorities for experimental screening.

2.6 Representative Tier A candidates

Table S10. Three representative Tier A reporter-screening candidates, one per cell type, selected from the locked non-overlapping 90-parent nine-criterion design benchmark. Representative examples were selected according to a prespecified rule requiring Tier A status, one example per target cell, positive cross-model margin gain, reduced uncertainty, and no synthesis-risk flag. Margins are shown for the primary Malinois predictor and the held-out reviewer ensemble; all three achieved Pareto-frontier status and carry no synthesis-risk flags. Edit positions use 0-indexed coordinates within the 200-nt sequence window. Margin and uncertainty values are in log-fold-change units. GC is expressed as a fraction. All edits are substitutions within a budget of five nucleotides. Motif-level observations at edited positions are reported as testable sequence-grammar hypotheses for follow-up.

Property	K562 (chr7:127284707)		HepG2 (chr7:105701659)		SK-N-SH (chr13:23368564)	
	Parent	Edited	Parent	Edited	Parent	Edited
Edit budget	5		5		5	
Edit positions	89, 118, 130, 132, 180		89, 94, 99, 112, 199		1, 148, 149, 171, 174	
Edit details	89T>A; 118C>G; 130A>T; 132C>A; 180C>A		89G>T; 94C>A; 99G>C; 112G>C; 199C>G		1C>G; 148T>C; 149C>T; 171A>G; 174C>A	
Primary margin	−0.372	+4.204	−0.620	+5.100	+0.020	+2.874
Reviewer margin	−0.138	+0.002	−0.068	+0.003	−0.197	−0.097
Primary margin gain	+4.577		+5.719		+2.854	
Reviewer margin gain	+0.140		+0.070		+0.100	
Primary target gain	+4.558		+6.093		+3.427	
Reviewer uncertainty	1.253	1.099	1.243	1.143	0.552	0.445
Uncertainty change	−0.154		−0.100		−0.107	
GC content	0.575	0.565	0.570	0.560	0.365	0.365
Naturalness Δ	+0.056		+0.006		+0.028	
Max homopolymer	4	4	5	5	5	5
Synthesis risk	None		None		None	
Pareto frontier	Yes		Yes		Yes	
Parent sequence	TGAGGGCAGAGCTCAGCCGCAACCCGGCCCTGCTGCTTAGAGGCTCGGGGCCTTGGCAAATGACAGCCTCCCTGAGCCTGTTTCTCCATCCGTAAAACAG<br>GCCTCCCAGCCCTGTGTCGGAGCAACTGAAACGAGGGTTAAATGAGGAAACCATGCGACAGATGTCAGCATAATCCCTGCCACGCAGAGGAGGCAAAATC					
Edited sequence (K562)	TGAGGGCAGAGCTCAGCCGCAACCCGGCCCTGCTGCTTAGAGGCTCGGGGCCTTGGCAAATGACAGCCTCCCTGAGCCTGTTTCTCCAACCGTAAAACAG<br>GCCTCCCAGCCCTGTGTGGGAGCAACTGATAAGAGGGTTAAATGAGGAAACCATGCGACAGATGTCAGCATAATCCCTGACACGCAGAGGAGGCAAAATC					
Parent sequence (HepG2)	GGGGGTGAGGGATCCCTGCAATACATAACACGTGCTAAGCAGTGGGGTTTGGGTGCTACTTCCCAACTGGCCGCTCTTAGGAACTGGTGAAACCTTAAGT<br>CAGGTGTGACTGAGCGAGCCCCTGGGTCATGGCTATTCTCCTTGCTGCTCTGCTCTCCTAAGTCTGGTTTGAGGCTCCTCTCCCTCCCTGGCGTCCCACC					
Edited sequence (HepG2)	GGGGGTGAGGGATCCCTGCAATACATAACACGTGCTAAGCAGTGGGGTTTGGGTGCTACTTCCCAACTGGCCGCTCTTAGGAACTGGTTAAACATTAACT<br>CAGGTGTGACTCAGCGAGCCCCTGGGTCATGGCTATTCTCCTTGCTGCTCTGCTCTCCTAAGTCTGGTTTGAGGCTCCTCTCCCTCCCTGGCGTCCCAGC					
Parent sequence (SK-N-SH)	CGAAATTCCCACAACTGGAATATATACCGCATTGATACTATCTTCCCCTTTATCCTAAAACTGTCATCTTTAAGCTTCAAACAGTACTGTGATTTGATGTT<br>AACCATGATGATTTGTACAACCGTATTTACAAGGCCACAACTCAAGTCATTTTTAGATTTCATCCTTCTAGACTGTAGCCAAATTCCCATCCATAAAGC					

Property	K562 P	K562 E	HepG2 P	HepG2 E	SK-N-SH P	SK-N-SH E
Edited sequence (SK-N-SH)	GGAAATTCCCACAACTGGAATATATACCGCATTGATACTATCTTCCCCTTTATCCTAAAACTGTCATCTTTAAGCTTCAAACAGTACTGTGATTTGATGTT<br>AACCATGATGATTTGTACAACCGTATTTACAAGGCCACAACTCAAGCTATTTTTAGATTTCATCCTTCTGGAATGTAGCCAAATTCCCATCCATAAAGC					

Note: P = parent natural CRE; E = SafeEdit-CRE edited design. Primary margin and target gain are from the Malinois predictor used to direct the search; reviewer margin and uncertainty are from the independent residual-dilated ensemble used during cross-model reranking. All edited sequences retain length 200 nt and exact edit budget. The full 74-candidate Tier A library is provided as a machine-readable table in the permanent Zenodo archive (v1.1.0).

3 Supplementary methods and thresholds

3.1 Ensemble training details, thresholds, and criteria definitions

Table S11. Training details and seeds for the reviewer and cross-model evaluation ensembles. Both ensembles were trained exclusively on the 627,661 training-split records.

Parameter	Reviewer ensemble	Cross-model evaluation ensemble	Common setting
Architecture	Residual-dilated 1D CNN (11-nt stem; dilations 1, 2, 4, 8)	Multi-kernel CNN (parallel 3-, 7-, 15-, 21-nt branches; pointwise fusion; depthwise conv.)	–
Seeds	20260721–20260723	20260731–20260733	3 members per ensemble
Training records	627,661	627,661	Training-split only
Max epochs	20	20	Early stopping: validation mean Pearson, patience 6
Optimizer	AdamW	AdamW	Cosine LR decay
Batch size	256	256	–
Learning rate	10^{-3}	10^{-3}	Weight decay 10^{-4}
Dropout	0.25	0.25	Gradient clipping 5.0
Loss	Heteroscedastic Gaussian NLL + reported measurement SE + RC-consistency penalty (weight 0.1)	Same	–
Test-split use	None	None	Never used for checkpoints, architectures, hyperparameters, or variance scales

Table S12. Documentation of all parameters and thresholds, their type, and the point at which each was fixed. Quantile thresholds are budget-specific; values below are reported per edit budget b in the order $b = 1/5/10/20$. The 600-parent rerank limits are the constants printed by the calibration step in the archived run log (2026-07-14); the 90-parent audit thresholds are documented in Table S13.

Parameter	Value	Type	Calibration source / locking
Strand-disagreement limit, 600-parent rerank	0.457 / 0.508 / 0.588 / 0.602	Data-driven ($q_{0.95}$)	Random edits of validation-split parents (96 parents); frozen 2026-07-14
Reviewer-uncertainty limit, 600-parent rerank	0.928 / 0.964 / 1.061 / 1.122	Data-driven ($q_{0.95}$)	Same
Absolute GC-change limit, 600-parent rerank	0.005 / 0.020 / 0.025 / 0.040	Data-driven ($q_{0.95}$)	Same
Naturalness-change limit, 600-parent rerank	-0.049 / -0.121 / -0.201 / -0.334	Data-driven ($q_{0.05}$)	Same
Audit thresholds, 90-parent benchmark	see Table S13	Data-driven	Independent calibration on random edits of 24 validation parents; see Table S13 note
Reranking weight: reviewer margin	0.3	Fixed constant	Chosen a priori; sensitivity via complete re-search ablation
Reranking weight: strand disagreement	0.1	Fixed constant	Same
Reranking weight: uncertainty	0.05	Fixed constant	Same

Parameter	Value	Type	Calibration source / locking
Generation prefilter: homopolymer	Discard runs > 8	Fixed constant	Synthesis feasibility at generation
Generation prefilter: GC change	Discard $\lvert\Delta GC\rvert > 0.12$	Fixed constant	Same
Rerank gate: homopolymer	Require runs ≤ 7	Fixed constant	Staged tightening
Audit: homopolymer	Require runs ≤ 6	Fixed constant	Final release criterion
Beam width	24	Fixed constant	Compute-matched control
Pre-screen size	$\min(24 \times 6, 96)$	Fixed constant	Same expansion schedule as control
Near-Pareto tolerance	5% relative / 0.1 absolute	Fixed constant	Reference frontier from primary–reviewer objectives
Benchmark seed	20260714	Fixed	Frozen configuration
Bootstrap seed / iterations	20260713 / 100,000	Fixed	Parent-cluster resampling

Table S13. Formal definitions of the nine Boolean design-quality checks and the audit thresholds. The four quantile-based thresholds were calibrated per edit budget from random edits of 24 validation-split parents, independently of the 600-parent rerank limits documented in Table S12, and were locked before any confirmatory parent was evaluated. Thresholds are shown per budget b in the order $b = 1/5/10/20$; each quoted range x–y brackets the exact threshold, which was reconstructed from the pass/fail boundaries of all 3,240 released frozen candidate rows but the exact constants were not archived with the public release; the quoted ranges are therefore the tightest bounds recoverable from the frozen candidate table, and only the values in this table can be recomputed from the released data. Rerunning the associated calibration would require the pilot-stage compact CNN reviewer checkpoint, which is not archived. The reviewer-based checks use the pilot-stage compact CNN ensemble reviewer of the confirmatory phase, a `HeteroscedasticCRECNN` architecture trained on the training partition only. Its predictive-uncertainty scale differs from that of the G7 residual-dilated reviewer used during 600-parent search, but each calibration is internally consistent with its own reviewer. All editing parents reside in the test partition, disjoint from either reviewer's training data.

Check	Formal criterion	Threshold ($b = 1/5/10/20$)	Calibration
Primary target gain non-negative	$\Delta T_{\text{target}} \geq 0$	fixed at 0	Fixed by design
Primary margin gain positive	$\Delta M_{\text{primary}} > 0$	fixed at 0	Fixed by design
Reviewer margin transfer positive	$\Delta M_{\text{reviewer}} > 0$	fixed at 0	Fixed by design
Strand disagreement in domain	$D_{\text{strand}} \leq q_{0.95}(b)$	0.893–0.914 / 0.754–0.763 / 0.583–0.584 / 0.849–0.855	Validation random edits, per budget
Reviewer uncertainty in domain	$U \leq q_{0.95}(b)$	2.290–2.543 / 2.511–2.588 / 2.597–2.625 / 2.513–2.540	Validation random edits, per budget
Naturalness change in domain	$\Delta N \geq q_{0.05}(b)$	-0.047–-0.046 / -0.104–-0.101 / -0.175–-0.173 / -0.304–-0.295	Validation random edits, per budget
GC change in domain	$\lvert\Delta GC\rvert \leq q_{0.95}(b)$	≥0.005 / 0.015–0.020 / 0.025–0.030 / 0.040	Validation random edits, per budget
Homopolymer safe	Max run ≤ 6	fixed at 6	Fixed by design
Exact edit budget	$d_H(x_{\text{edit}}, x_{\text{parent}}) = b$	exact	Fixed by design

3.2 Rejection decomposition and primary-rank acceptance

Table S14. Nine-criterion rejection decomposition in the 90-parent confirmatory benchmark, 1,080 designs per method. Entries are numbers of failures per criterion; values in parentheses count the designs for which the criterion was the sole failing one.

Criterion	SafeEdit-CRE	Greedy	Random
Primary target gain non-negative	64 (33)	57 (28)	535 (88)
Primary margin gain positive	3 (0)	0 (0)	509 (68)
Reviewer margin transfer positive	302 (252)	352 (204)	540 (126)

Criterion	SafeEdit-CRE	Greedy	Random
Strand disagreement in domain	13 (2)	221 (112)	25 (8)
Reviewer uncertainty in domain	10 (2)	18 (3)	13 (0)
Naturalness change in domain	11 (4)	43 (16)	60 (7)
GC change in domain	7 (3)	86 (33)	60 (9)
Homopolymer safe	103 (64)	123 (60)	112 (25)
Exact edit budget	0 (0)	0 (0)	0 (0)
Accepted (all nine criteria)	654	419	142
Infeasible designs	0	0	0

Table S15. Nine-criterion acceptance rate by quartile of the primary (Malinois) candidate-margin rank, computed within each target–budget stratum. Q1 is the lowest-margin quartile.

Method	Rank quartile	n	Passed	Acceptance (%)
SafeEdit-CRE	Q1 (low)	276	177	64.1
SafeEdit-CRE	Q2	264	155	58.7
SafeEdit-CRE	Q3	264	163	61.7
SafeEdit-CRE	Q4 (high)	276	159	57.6
Greedy Malinois	Q1 (low)	276	97	35.1
Greedy Malinois	Q2	264	97	36.7
Greedy Malinois	Q3	264	112	42.4
Greedy Malinois	Q4 (high)	276	113	40.9
Random matched	Q1 (low)	276	20	7.2
Random matched	Q2	264	27	10.2
Random matched	Q3	264	44	16.7
Random matched	Q4 (high)	276	51	18.5

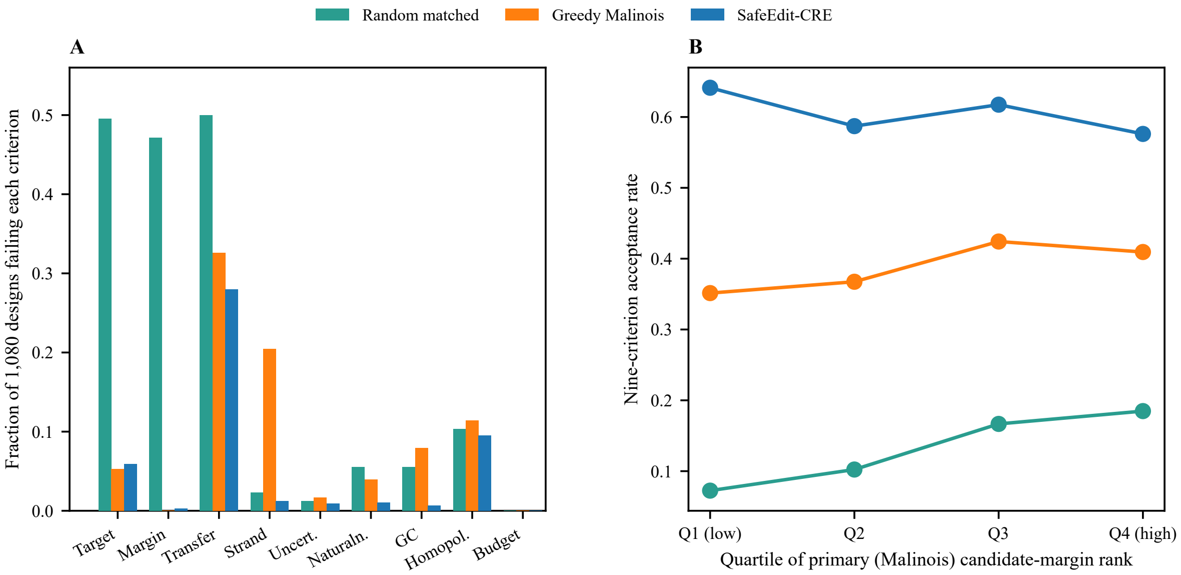

Figure S4. Rejection decomposition and primary-rank acceptance. **A**, fraction of 1,080 designs per method failing each of the nine criteria. Abbreviations: Target = non-negative primary target gain; Margin = positive primary margin gain; Transfer = positive reviewer margin transfer; Strand = strand disagreement within domain; Uncert. = reviewer uncertainty within domain; Naturaln. = naturalness within domain; GC = GC change within domain; Homopol. = homopolymer safety; Budget = exact edit budget. **B**, nine-criterion acceptance rate by quartile of the primary candidate-margin rank (Q1 lowest).

Table S16. Best-of-three sensitivity analysis for random matched editing in the 600-parent evaluation, computed from the per-replicate records (seeds 20260801–20260803; 7,200 parent–target–budget conditions). Best-of-three takes the per-condition maximum for gain endpoints, the minimum for uncertainty as the favorable direction, and the union rule (any replicate positive) for the positive-margin fraction. Under this favorable selection the random margin and target gains remain an order of magnitude below the directed methods. The lower best-of-three uncertainty reflects that random edits change the sequence minimally and therefore incur little predictive change but almost no specificity gain.

Endpoint	Averaged (3 seeds)	Best-of-3	SafeEdit-CRE
Cross-model margin gain	0.001	0.105 (95% CI 0.099–0.110)	0.877
Cross-model target gain	0.012	0.250	1.661
Positive-margin fraction (%)	48.6	85.8	95.4
Cross-model uncertainty	0.185	0.125	0.314

4 Reproducibility summary

Parent selection was deterministic and label-blind, with all historical identifiers, sequence hashes, and audit-flagged conflicts removed before benchmark construction. The post-freeze evaluator was loaded only after candidate files and their SHA-256 manifests had been written. The compute-matched beam used the same beam width, expansion depth, and pre-screen size as SafeEdit; random comparisons averaged three fixed replicates. Final confidence intervals used 100,000 parent-cluster bootstrap replicates. All seven ablation configurations reran the complete search, and every reported figure and table can be regenerated from the candidate-level data included with the analysis scripts.
